# Molecular Approaches for the Validation of the Baboon as a Nonhuman Primate Model for the Study of Zika Virus Infection

**DOI:** 10.3389/fcimb.2022.880860

**Published:** 2022-04-14

**Authors:** Emma Mask, Vida L. Hodara, Jessica E. Callery, Laura M. Parodi, Veronica Obregon-Perko, Shigeo Yagi, Jeremy Glenn, Patrice Frost, Elizabeth Clemmons, Jean L. Patterson, Laura A. Cox, Luis D. Giavedoni

**Affiliations:** ^1^ Department of Biology, Trinity University, San Antonio, TX, United States; ^2^ Southwest National Primate Research Center, San Antonio, TX, United States; ^3^ Texas Biomedical Research Institute, San Antonio, TX, United States; ^4^ California Department of Public Health, Richmond, CA, United States; ^5^ Center for Precision Medicine, Wake Forest Health Sciences University, Winston Salem, NC, United States

**Keywords:** transcriptomic, immunology and infectious diseases, cytokines, nonhuman primate (NHP), arboviral diseases

## Abstract

Nonhuman primates (NHP) are particularly important for modeling infections with viruses that do not naturally replicate in rodent cells. Zika virus (ZIKV) has been responsible for sporadic epidemics, but in 2015 a disseminated outbreak of ZIKV resulted in the World Health Organization declaring it a global health emergency. Since the advent of this last epidemic, several NHP species, including the baboon, have been utilized for modeling and understanding the complications of ZIKV infection in humans; several health issues related to the outcome of infection have not been resolved yet and require further investigation. This study was designed to validate, in baboons, the molecular signatures that have previously been identified in ZIKV-infected humans and macaque models. We performed a comprehensive molecular analysis of baboons during acute ZIKV infection, including flow cytometry, cytokine, immunological, and transcriptomic analyses. We show here that, similar to most human cases, ZIKV infection of male baboons tends to be subclinical, but is associated with a rapid and transient antiviral interferon-based response signature that induces a detectable humoral and cell-mediated immune response. This immunity against the virus protects animals from challenge with a divergent ZIKV strain, as evidenced by undetectable viremia but clear anamnestic responses. These results provide additional support for the use of baboons as an alternative animal model to macaques and validate omic techniques that could help identify the molecular basis of complications associated with ZIKV infections in humans.

## Introduction

Zika virus (ZIKV), a positive sense single-stranded RNA virus, belongs to the *Flaviviridae* family, which includes other viruses such as the yellow fever, dengue, and West Nile viruses. ZIKV was initially discovered in the serum of a febrile sentinel Rhesus macaque monkey in the Zika forest of Uganda in 1947. During the 2013 ZIKV epidemic in the French Polynesia, an increased incidence of Guillain-Barré syndrome (GBS) was observed in adults, and an increased incidence of microcephaly and brainstem dysfunction was observed in fetuses and newborns (Saiz et al., 2017). Despite this, research on ZIKV remained relatively neglected up until the 2015 Brazil outbreak, which exhibited similar increases in GBS and neurological complications in fetuses and newborns (Wikan and Smith, 2016; Bradley and Nagamine, 2017; Saiz et al., 2017; Dong and Liang, 2018). Consequently, in February of 2016, the World Health Organization declared ZIKV a global health emergency (Dong and Liang, 2018). As clear from the current COVID-19 pandemic and frequent episodes of flu and Ebola virus epidemics, viral diseases represent the most immediate infectious disease threat to global human health. In that context, reliable animal models remain critical for understanding pathogenesis of viral infection and for the development of vaccines and therapeutic approaches to limit their impact in human populations. Based on close similarities in genomic organization, physiology, and susceptibility to viral infections, nonhuman primate (NHP) models are recognized as the most translational animal models (Estes et al., 2018).

Different NHP models have been developed to address issues such as ZIKV pathogenesis and vaccine development. Asian macaque species, including rhesus macaques [*Macaca mulatta*, (Dudley et al., 2016; Li et al., 2016; Osuna et al., 2016; Carroll et al., 2017)], cynomolgus macaques [*M. fascicularis*, (Koide et al., 2016; Osuna et al., 2016)], and pig-tailed macaques [*M. nemestrina*, (Adams Waldorf et al., 2016)], were the first nonhuman species used for modeling ZIKV infections. Other models included a New World primate species such as the marmoset [*Callithrix jacchus*, (Chiu et al., 2017)], and African species such the African green monkey [*Chlorocebus sabaeus*, (Haddow et al., 2020)] and the olive baboon [*Papio anubis*, (Gurung et al., 2018)], due to their likely role as a zoonotic reservoir for ZIKV (Buechler et al., 2017).

The olive baboon model of ZIKV infection has an important translational value, considering that baboons resemble humans closely with respect to size, genetics (Cox et al., 2013), placenta formation (Carter, 2007), reproduction (Kyama et al., 2007; Li et al., 2018), brain development (Banks et al., 2017), immunology (Attanasio et al., 2002; Giavedoni et al., 2004; Warfel and Merkel, 2014), and potential for stem-cell modification (Navara et al., 2018; Olivier et al., 2019). Previous studies of infection of baboons with ZIKV have shown similarities of clinical signs (Gurung et al., 2018), transmission of ZIKV from infected dams to their fetuses with considerable fetal lesions (Gurung et al., 2019), transient presence of ZIKV in the semen of infected males (Peregrine et al., 2019), and transmission of ZIKV infection to female baboons after exposure to ZIKV-infected baboon semen (Gurung et al., 2020). In this study, we validate the baboon model with the use of molecular and immunological tools. We show that the acutely infected baboon, even in the absence of noticeable clinical signs, presents molecular signatures of antiviral responses similar to the ones observed in ZIKV-infected humans. This weak immune response is enough to protect animals against a re-challenge with a divergent ZIKV.

## Materials and Methods

### Animal Ethics Statement

All animal studies were conducted at the Southwest National Primate Research Center (SNPRC), Texas Biomedical Research Institute; molecular, viral, and transcriptome analyses of baboon body fluids and tissues were conducted at Texas Biomed. Texas Biomed is accredited by the Association for Assessment and Accreditation of Laboratory Animal Care (AAALAC) International and operates in accordance with the NIH and U.S. Department of Agriculture guidelines and the Animal Welfare Act. The Institutional Animal Care and Use Committee (IACUC) and the Institutional Biohazards Committee (IBC) of Texas Biomed approved all baboon experiments related to this study. All experiments were performed in accordance with relevant guidelines and regulations.

### Animal Studies

Four juvenile baboons (32535, 32569, 32588, and 32753) were kept healthy and well-nourished with strict feeding protocols and close monitoring of their health status prior to the start of the study and during the entire study period. One week before inoculation, animals were transferred to the biosafety level (BSL)-2+ facility at the SNPRC and housed individually in cages. As they are social animals in the wild, all baboons had auditory, visual, and olfactory access to each other throughout the study. At the end of the study, baboons were sedated and humanely euthanized by administration of a sodium pentobarbital solution by a licensed veterinarian at the SNPRC. Baboons were injected with 10^4^ (32569 and 32753) or 10^6^ (32535 and 32588) TCID_50_ of the Puerto Rico strain of ZIKV (PRVABC59, 2015) by the subcutaneous route in the interscapular region. Six months after primary infection (week 25 post-ZIKV PR2015 challenge), the same animals were exposed to 10^4^ TCID_50_ of the Uganda strain of ZIKV (ZIKV UG1947). ZIKV strains were obtained from the Viral and Rickettsial Disease Laboratory (VRDL) branch of the California Department of Public Health and were amplified by one additional passage on Vero cells (Chiu et al., 2017). Animals were sedated at days -7, 0, 1, 3, 6, 9, 13, 15, 17, 21, and 28 after each viral exposure. Biological samples obtained from experimental animals consisted of blood, mucosal (oral and rectal) swabs, and urine; systemic tissues were collected after necropsy. During each sedation, animals were evaluated for body weight, heart and respiratory rate, presence of abdominal skin rash and conjunctivitis, and measurement of rectal temperature.

### Measurement of ZIKV RNA Loads by Quantitative RT-PCR

The course of infection in inoculated animals was monitored by determination of ZIKV RNA loads (expressed as RNA copies/mL) in plasma, urine, and mucosal swabs. Estimated ZIKV RNA loads were calculated by generation of a standard curve, followed by quantitative RT-PCR testing for 40 cycles using primers targeting the envelope gene (ZIKV-1086/ZIKV-1162). By standard curve analysis, the estimated limit of detection for the qRT-PCR assay is ~15 RNA copies/mL).

### Lymphocyte Phenotyping and Activation State

Phenotypic characterization of baboon peripheral blood mononuclear cells (PBMCs) was performed by multicolor flow cytometry using direct immunofluorescence. Aliquots of 100 μl of EDTA whole blood were directly incubated with antibodies for 20 minutes at room temperature; red blood cells were lysed with ammonium-chloride-potassium (ACK) buffer, and cells were then washed twice with phosphate-buffered saline (PBS) and fixed with 1.6% methanol-free formaldehyde before analysis in a CyAn ADP flow cytometer (Beckman-Coulter). The antibodies used for this analysis were conjugated to fluorescein isothiocyanate (FITC), Phycoerythrin (PE), Peridinin-chlorophyll-cyanin 5.5 (PerCP-Cy5.5), Phycoerythrin-cyanin 5.1 (PC5), Phycoerythrin-cyanin 7 (PC7), Pacific Blue, BD Horizon V500, Allophycocyanin (APC) or Alexa Fluor 700. Antibodies included in this study were: CD3 (clone SP34.2), CD4 (clone L200), CD154 (clone TRAP-1), and HLA-DR (clone G46.6/L243) from BD-Biosciences; CD14 [clone 322A-1 (My4), CD159a (NKG2A; clone Z199), CD20 (clone H299(B1)], CD335 (NKp46; clone BAB281) and CD337 (NKp30; clone Z25) from Beckman-Coulter; CD16 (clone 3G8), CD8 (clone 3B5), CD40 (clone 5C3), CD83 (clone HB15), and CD86 (clone IT2.2) from Biolegend; and CD159c (NKG2C;clone 134522) from R&D Systems. For analyses, lymphocytes were gated based on their characteristic forward and side scatter pattern, followed by T-cell selection using a second gate on the CD3-positive population. Thus, CD8 T cells were defined as CD8^+^/CD3^+^ and CD4 T cells as CD4^+^/CD3^+^. Natural Killer cells (NK) were defined as CD3^-^/CD20^-^/CD14^-^ lymphocytes and analyzed by the expression of NK cell markers CD16, CD8, NKG2A, NKG2C, NKp30 and NKp46. B cells were defined as CD20+/CD3^-^/CD14^-^.

### Multiplex Cytokine Analysis of Plasma Samples

Plasma samples were analyzed for baboon cytokines and chemokines on the Luminex 200 system using established protocols for baboon biomarkers (Reyes et al., 2017; Obregon-Perko et al., 2018; Scordo et al., 2021). The assay included evaluation of the following 25 analytes: B-cell activating factor (BAFF), growth-related oncogene-α (GRO-α; CXCL1), interferon alpha (IFN-α), IFN-γ, interferon γ-induced protein 10 kDa (IP-10, CXCL10), interleukin-1 beta (IL-1β), IL-1 receptor antagonist (IL-1RA), IL-4, IL-8, IL-10, IL-12 p40 and p70, IL-15, IL-18, IL-22, monocyte chemoattractant protein 1 (MCP-1, CCL2), macrophage migration inhibitory factor (MIF), monokine induced by gamma interferon (MIG, CXCL9), macrophage inflammatory protein 1-alpha (MIP-1α, CCL3), MIP-1β (CCL4), regulated on activation, normal T cell expressed and secreted (RANTES, CCL5), tumor necrosis factor-alpha (TNF-α), soluble CD40 ligand (sCD40L), soluble intercellular adhesion molecule 1 (sICAM-1), and vascular endothelial growth factor A (VEGF-A).

### ZIKV Serological Analysis by Antibody Neutralization

Plaque-reduction neutralization testing (PRNT) on longitudinally collected baboon plasma was performed by the California Department of Public Health. The protocol was similar to that used by the US CDC for confirmatory ZIKV testing in patients (Chiu et al., 2017). Briefly, 100 plaque forming units (PFU) of ZIKV (1947 Uganda strain or 2015 PR strain, depending on the strain that was inoculated) were mixed with equal volumes of serial 2-fold dilutions of inactivated baboon plasma and incubated for 1 hr at 36°C, followed by addition of this mixture to a monolayer culture of Vero cells for 1 hr at 36°C. After removal of the inoculum, 3 mL of 2% agar in Eagle’s Minimal Essential Medium (MEM) were added, plates were placed in a 36°C, 5% CO_2_ incubator for 3 days, followed by addition of 3 mL of 1% agar and 0.004% neutral red in Eagle’s MEM and another 1–2 days of incubation until plaques were formed. An 80% reduction of the number of plaques compared to positive control wells inoculated with virus-diluent mixtures was considered neutralization, with serum titers reported as the highest dilution exhibiting ≥80% reduction.

### ELISA for ZIKV-Binding Antibodies

ELISA assays for ZIKV-binding antibodies on longitudinally collected baboon plasma samples were performed with commercially available kits. Cross-reacting baboon anti-Dengue Virus envelope IgG antibodies were identified with the Dengue Virus Igg Dxselect kit (DiaSorin Molecular, Cat. No. El1500g), whereas anti-ZIKV NS1 binding IgG antibodies were detected with the Recombivirus Monkey Anti-Zika Virus NS1 IgG ELISA Kit (Alpha Diagnostic International, Cat. No. RV-403310). Binding was expressed as optical density at 450 nm.

### ZIKV-Specific Cell-Mediated Responses

Detection of ZIKV-specific baboon cell-mediated immune responses was performed with a modified indirect T cell recognition assay (ITRA, (Tong et al., 2014). Briefly, baboon PBMC were obtained from anticoagulated blood and resuspended in RPMI with 10% FBS (RPMI-10) at 10^7^ cell/ml. Aliquots of 100 μl of cell suspension were added to each of three 4-ml polypropylene tubes labeled NEG, POS, and ZIKV. The NEG tube received 100 μl of supernatant from HEK cells stably transduced with an empty lentiviral vector. The POS tube received 100 μl of *Staphylococcus* enterotoxin A/B at 2 μg/ml in RPMI-10. The ZIKV tube received 100 μl of supernatant from HEK cells stably transduced with a lentiviral vector expressing the ZIKV prME gene. Cells were incubated for 24 hs at 37°C and then centrifuged to separate cells and supernatant. Cells were stained for expression of CD3, CD4, CD8, CD20, CD40, CD83, and CD154 as detailed in the Lymphocyte Analysis section, while supernatants were analyzed by Luminex for measurement of the concentration of CXCL-9/MIG, CXCL10/IP10, CXCL-11/I-TAC, IL-2, IL-6, IFN-γ, perforin, and TNF-α. Cytokine production and lymphocyte activation values for ZIKV activation versus control wells for the four baboons were analyzed by two-tailed paired t-tests and considered significant if P values were smaller than 0.05.

### RNA Sequencing

RNA was isolated from PBMCs using the Direct-zol™ RNA Microprep Kit (Zymo Research), then quantitated by Qubit fluorometric assay (ThermoFisher Scientific) and quality assessed by Agilent Tapestation (Agilent Technologies). 50-100 ng of high-quality RNA per sample was then normalized by concentration to perform library preparation. The KAPA mRNA HyperPrep Kit (Roche) was used to construct strand-specific, uniquely indexed cDNA libraries for multiplexed sequencing. To prepare libraries of mRNA transcripts, each RNA sample was subjected to mRNA capture using magnetic oligo-dT beads, fragmentation using heat and magnesium, random primed 1^st^ strand cDNA synthesis, combined 2nd strand cDNA synthesis and A-tailing, barcode adapter ligation, and library amplification using high-fidelity, low-bias PCR. Agilent Tapestation (Agilent Technologies) was used to assess cDNA library quality and fragment size. The number of fragments carrying appropriate adapter sequences at both ends were quantified by qPCR on a QuantStudio 5 Real-Time PCR system (ThermoFisher Scientific) using the KAPA Library Quantification kit for Illumina platforms (Roche). Individual libraries were then normalized by concentration and pooled according to compatible indices prior to sequencing. Transcripts were sequenced using the Illumina HiSeq 2500 platform, which also performed primary analyses of the raw sequence data, including calling, quality filtering, index de-multiplexing, and adapter trimming. These primary analyses provided approximately 10 million high-quality, 100 bp paired end reads per sample in FASTQ format, which were then used for secondary data analyses performed with a pipeline of software tools available from Partek Flow (Partek Inc.). These secondary analyses included steps such as additional filtering of reads to base quality of Phred 30, and alignment of reads to the publicly available assembly of the olive baboon genome, Panubis 1.0 (Batra et al., 2020), with the RNA-Seq aligner pipeline STAR 2.5.3a (Dobin et al., 2013), post alignment quality assessment, and transcript abundance estimation according to Ensembl transcriptome annotation by expectation- maximization algorithm.

### Transcriptomic Analysis

Principal component analysis (PCA) plots were generated using the R/Bioconductor package DESeq2 [version 1.34.0, RRID: SCR_015687 (Love et al., 2014; RStudio Team, 2018; R Core Team, 2019)], and indicated that one of the four animals (32535) was an outlier at day 0 post-infection (**Supplemental Figure 1A**). A second PCA plot was generated excluding data from animal 32535 at day 0, and indicated that animal 32535 was also an outlier at the other two timepoints included in the RNA-seq data (days 3 and 15 post-infection) (**Supplemental Figure 1B**). Due to these results, all data from animal 32535 were excluded from downstream analyses.

Data normalization and differential expression (DE) analysis of gene count data were also performed using the R/Bioconductor DESeq2 package [version 1.34.0, RRID: SCR_015687 (Love et al., 2014; RStudio Team, 2018; R Core Team, 2019)]. Pairwise analyses were conducted to calculate the fold-change in expression of genes between 3 *vs* 0 dpi, 15 *vs* 0 dpi, and 15 *vs* 3 dpi.

Gene Set Enrichment Analysis (GSEA) (version 4.2.2, RRID: SCR_003199) was performed using the hallmark and Reactome canonical pathways gene sets from the Molecular Signatures Database (MSigDB) (version 7.5.1). The Database for Annotation, Visualization, and Integrated Discovery (DAVID) Functional Annotation tool [2021 Update, RRID: SCR_001881 (Huang et al., 2009a; Huang et al., 2009b)] was used to detect enriched Biological Process gene ontology (GO) terms. Gene lists for DAVID GO analyses were generated by filtering DE analysis results for genes with an FDR ≤ 0.05 and LFC ≥ ± 1.0.

## Results

### Virus Dynamics

**Figure 1:**
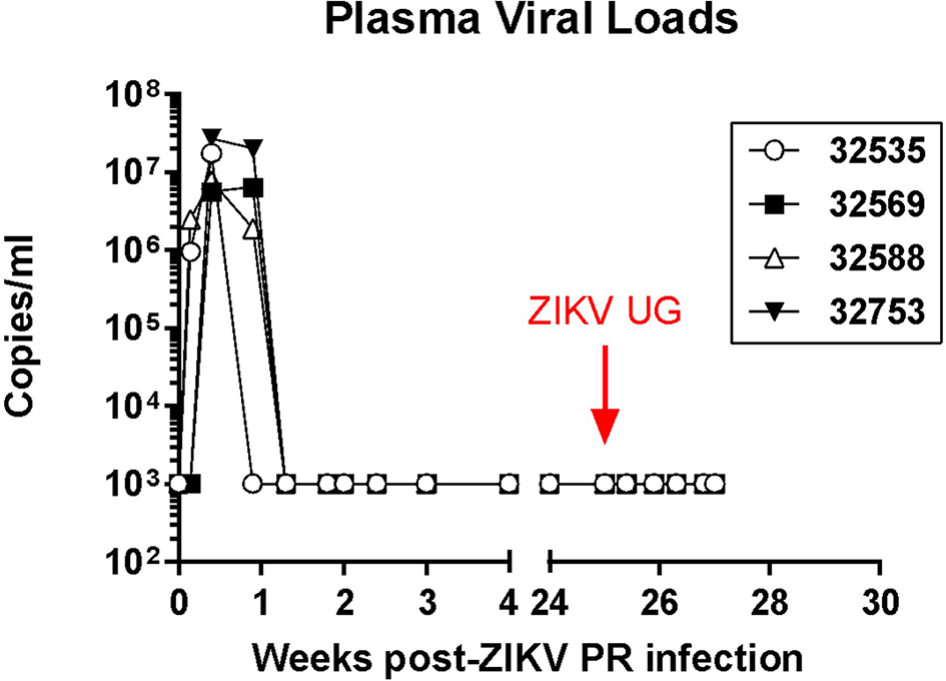
Plasma ZIKV loads in four olive baboons. The baboons were initially infected with the Puerto Rico strain of ZIKV (PRVABC59, 2015) *via* subcutaneous injection with the virus in either a high dose of 10^6^ TCID_50_ (*n* = 2, open symbols) or a low dose of 10^4^ TCID_50_ (*n* = 2, closed symbols). After 25 weeks, the baboons were exposed to 10^4^ TCID_50_ of the divergent ZIKV Uganda strain (ZIKV UG1947) through subcutaneous injection.

Baboons were inoculated by the subcutaneous route with two different doses of the Puerto Rico strain of ZIKV (PRVABC59, 2015), and blood, urine, and mucosal (oral and rectal) swab samples were collected every 2-3 days during the acute phase of infection. Animals that received the 10^6^ TCID_50_ dose became viremic as early as one day post-infection (dpi), while the animals receiving the 10^4^ TCID_50_ had detectable virus by 3 dpi (**Figure 1**). Viremia was present in all animals and lasted for about 5 days. Virus was sporadically present in the urine of some animals during this viremic period. There were no visible clinical signs of infection in any of the animals (including weight loss, body rash, and conjunctivitis), and body temperature was stable during the viremic phase. After a 25-week period, all animals were exposed to 10^4^ TCID_50_ of the divergent ZIKV Uganda strain (ZIKV UG1947) by the same SQ route. Samples taken from all animals during this post-challenge period followed a schedule similar to the one performed during the primary infection and were below the limit of detection by the same real time RT-PCR assay, indicating that re-infection was controlled in these convalescent animals (**Figure 1**). Back titration on Vero cells of both ZIKV stocks confirmed the correct dose of infectious viruses.

### Systemic Immune Changes After ZIKV Infection

**Figure 2:**
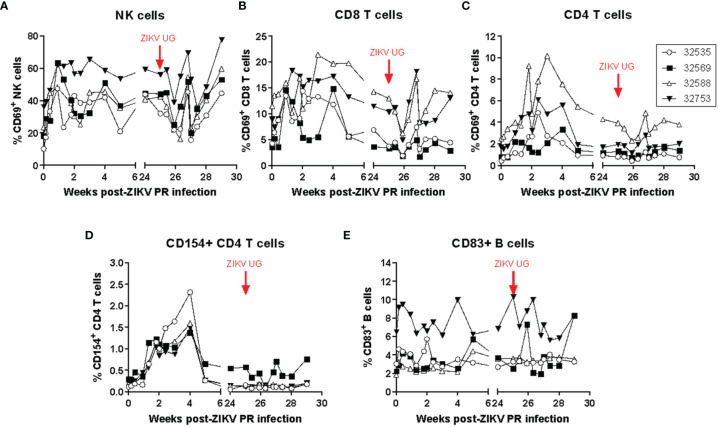
ZIKV infection in baboons induces a detectable transient systemic change in the activation state of NK, CD8 T, CD4 T, and B cells. The percentage of **(A)** NK cells and **(B)** CD8 T cells expressing CD69 increased immediately after infection with ZIKV PR. **(C)** The percentage of CD4 T cells expressing CD69 peaked at approximately 2 weeks post-infection. **(D)** The percentage of CD4 T cells expressing CD154 also increased after infection and peaked at 4 weeks post-infection. No changes in expression were detected after challenge with ZIKV UG. **(E)** Expression of CD83 on B cells. Activation markers were detected using polychromatic flow cytometry. The baboons were challenged with the virus in either a high dose of 10^6^ TCID_50_ (*n* = 2, open symbols) or a low dose of 10^4^ TCID_50_ (*n* = 2, closed symbols).

Systemic immune changes induced by acute infection and rechallenge with ZIKV were identified in blood samples obtained frequently after ZIKV infection. Changes in lymphocyte subset numbers and their activation state were monitored by polychromatic flow cytometry (**Figure 2**). While there were no significant changes in circulating lymphocyte subset numbers after ZIKV infection, the activation state of several cells increased during the acute stage of infection. Compared to pre-infection levels, the percentage of lymphocytes expressing the early activation marker CD69 increased immediately after primary infection on NK cells (**Figure 2A**) and CD8 T cells (**Figure 2B**), while it peaked at about 2 weeks post-infection for CD4 T cells (**Figure 2C**). Expression of the CD4 T cell activation marker CD154 (CD40L) also increased after infection and reached a peak of expression by 4 weeks -post infection; interestingly, there was no detectable change in the expression of this marker after challenge with ZIKV UG (**Figure 2D**). Expression of the activation marker CD83 on B cells was not consistent for all animals, although there was a detectable transient increase after the primary infection with ZIKV PR (**Figure 2E**). The evaluation of several NK activating and inhibitory receptors such as CD16, NKG2A, NKG2C, NKp30, and NKp46 did not result in any consistent and significant pattern in response to ZIKV infection (data not shown). This flow cytometry analysis suggests that even a subclinical ZIKV infection can induce detectable transient systemic changes in the activation state of certain lymphocyte subset.

**Figure 3:**
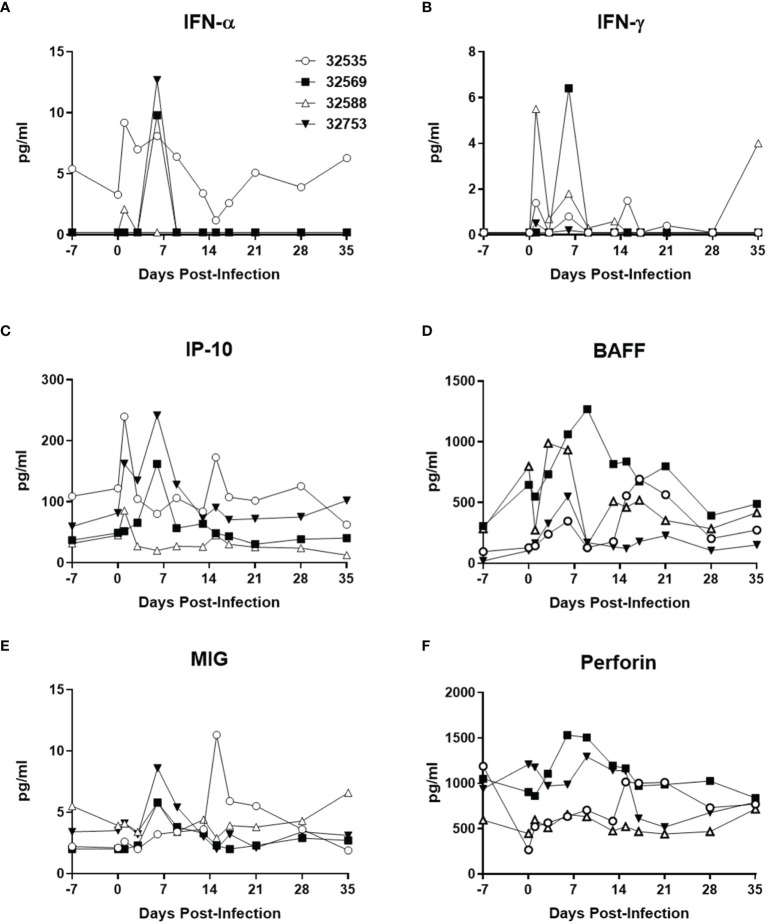
Baboon plasma cytokine levels after ZIKV PR infection. **(A)** IFN-α, **(B)** IFN-γ, **(C)** IP-10/CXCL10, **(D)** BAFF, **(E)** MIG/CXCL9, and **(F)** perforin. Cytokines were measured using Luminex assays. The baboons were challenged with the virus in either a high dose of 10^6^ TCID_50_ (*n* = 2, open symbols) or a low dose of 10^4^ TCID_50_ (*n* = 2, closed symbols).

Changes in the concentration of plasma cytokines were determined by Luminex analysis with 25 anti-human cytokine antibody pairs validated for baboon molecules (Giavedoni et al., 2004; Giavedoni, 2005; Hardy et al., 2009; Scordo et al., 2021; Singh et al., 2021) (**Figure 3**). The two baboons that were inoculated with 10^6^ TCID_50_ of ZIKV PR had detectable peaks of IFN-α by 1 day post-infection (dpi), whereas the animals receiving 10^4^ TCID_50_ of ZIKV PR peaked by 6 dpi (**Figure 3A**). A similar pattern of early and transient peaks was detected for IFN-γ (**Figure 3B**), and the IFN-γ-related cytokines IP-10 (**Figure 3C**) and MIG (**Figure 3E**). The tumor necrosis factor ligand family member BAFF, involved in B-cell activation and antibody production also showed an increase in concentration that peaked at around one week post-ZIKV PR infection (**Figure 3D**), whereas perforin levels followed a similar profile but with a smaller change of concentration (**Figure 3F**). None of these systemic changes observed after primary ZIKV PR infection were detected after challenge with ZIKV UG (**Supplemental Figure 2**). Combined with the observation of undetectable viremia, this suggests that the viral antigen load generated after ZIKV UG challenge was insufficient to elicit detectable cytokine release.

### Analysis of the Anti-ZIKV Immune Response

**Figure 4:**
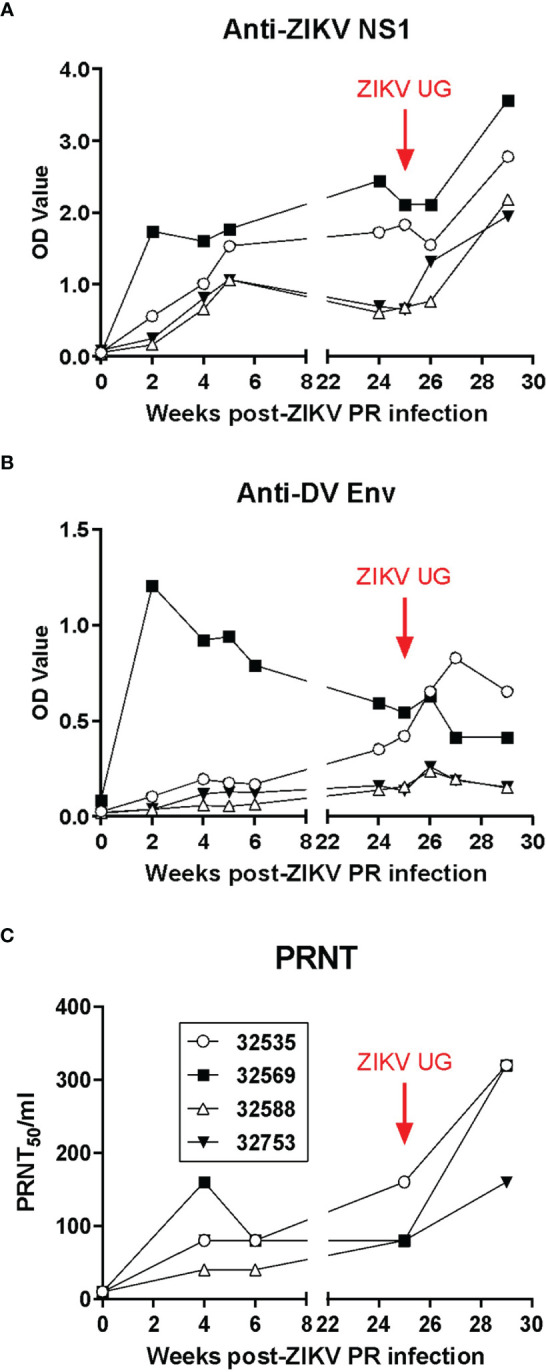
Humoral immune responses to ZIKV in exposed baboons. **(A)** ELISA anti-ZIKV NS1 IgG levels. **(B)** ELISA cross-reactive anti-Dengue virus GP IgG antibodies. **(C)** Plaque-reduction neutralization test (PRNT) on longitudinally collected baboon plasma. Baboons were challenged with the virus in either a high dose of 10^6^ TCID_50_ (*n* = 2, open symbols) or a low dose of 10^4^ TCID_50_ (*n* = 2, closed symbols).

The analysis of the anti-ZIKV humoral responses in baboons after primary infection and challenge was performed by both ELISA and plaque-reduction neutralization (PRNT) tests (**Figure 4**). ELISA assays identified the induction of baboon antibodies that bound to ZIKV nonstructural protein 1 (NS1, **Figure 4A**) or cross-reacted with Dengue virus envelope glycoprotein (**Figure 4B**). In general, there was no difference in magnitude of the antibody response in relation to the ZIKV challenge dose; in fact, baboon 32569, which was inoculated with 10^4^ TCID_50_ of ZIKV PR, developed a very rapid antibody response that peaked by 2 weeks post-infection. A similar pattern of response was observed for the generation of neutralizing antibodies in the infected baboons, with baboon 32569 having a significantly higher titer than the other 3 animals (**Figure 4C**). After challenge with ZIKV UG, anti-ZIKV NS1 and neutralizing antibodies significantly increased in titers, whereas cross-reacting anti-DV Env antibodies had a transient uptick in responses by one week-post challenge. These anamnestic antibody responses suggest that some level of viral replication, below the limit of detection of the real time RT-PCR assay, occurred and stimulated antibody production.

**Figure 5:**
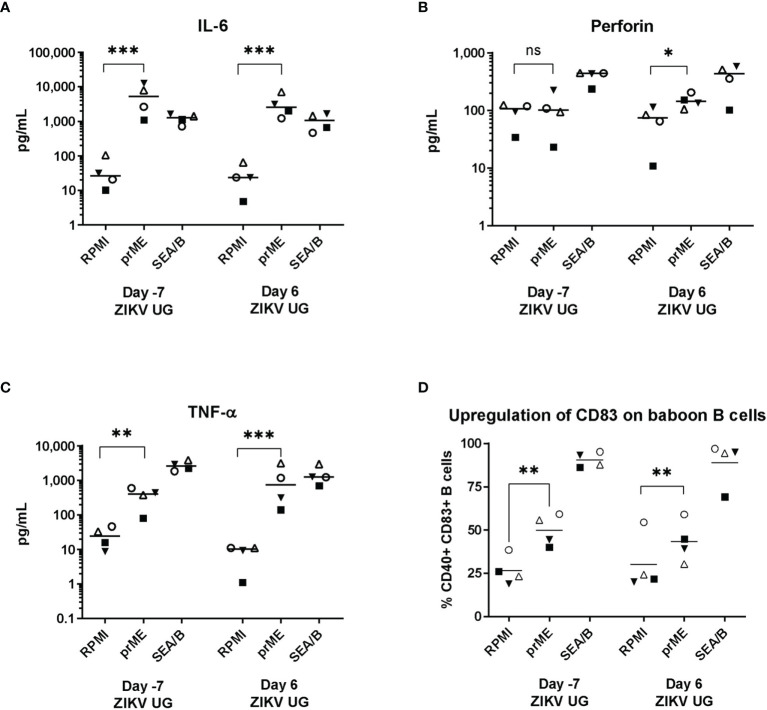
ZIKV-specific AIM assay. Baboon PBMCs were obtained a week before and a week after challenge with ZIKV UG, and were incubated with supernatant of HEK cells transduced with empty lentiviral vector (RPMI), or with supernatant of HEK cells transduced with a lentiviral vector expressing ZIKVprME protein (prME); *Staphylococcus* enterotoxin A and B (SEA/B) was used as positive control. After 24 hr stimulation supernatants were collected and used to measure **(A)** IL-6, **(B)** Perforin, **(C)** TNF-α with a Luminex assay. **(D)** Cells were used to measure CD83 expression on CD20 B cells. Two-tailed paired t-test. ns, not significant (p>0.05), * = p< 0.05; ** = p<0.01; *** = p<0.001.

The anti-ZIKV cell-mediated immune response was evaluated with an antigen-induced marker (AIM) assay that interrogated both the production of certain cytokines as well as the upregulation of cell surface markers on baboon cells exposed to ZIKV viral-like particles; assays were performed before and after challenge with ZIKV UG (**Figure 5**). Analysis of cytokines produced by ZIKV-stimulated baboon PBMC showed that expression of IL-6 was significant before challenge with ZIKV UG, almost after 6 months post-infection with ZIKV PR, and did not change significantly immediately after challenge with ZIKV UG (**Figure 5A**). Interestingly, expression of Perforin after ZIKV stimulation was only significantly different after challenge with ZIKV UG (**Figure 5B**), and this augmentation in cytokine production after challenge was also observed for TNF-α (**Figure 5C**). When analyzing upregulation of CD83 on B-cells in response to ZIKV stimulation, we observed a significant antigen-driven response, but responses of these baboon cells obtained before and after ZIKV UG challenge were of similar magnitude (**Figure 5D**). Data from this AIM assay demonstrate that primary ZIKV PR infection induces immunological memory in baboons that lasts at least 6 months, and that may become stimulated after re-exposure to the virus.

### Transcriptomic Analysis

We analyzed changes in the baboon PBMC transcription levels by RNAseq and differential expression analysis. We extracted PBMC RNA from the four baboons before infection with ZIKV PR (day 0), and on days 3 and 15 post-infection, which corresponded to the acute phase (day 3) and after resolution of infection (day 15). The average sequencing depth was 3.07 million reads per sample ( ± 1.04 M reads). Principal component analysis (PCA) plots indicated that one of the four animals (32535) was an outlier at day 0 post-infection (**Supplemental Figure 1A**). A second PCA plot was generated excluding data from animal 32535 at day 0 and indicated that animal 32535 was also an outlier at the other two timepoints included in the RNA-seq data (days 3 and 15 post-infection) (**Supplemental Figure 1B**). Due to these results, all data from animal 32535 were excluded from downstream analyses. Differential expression analyses detected 27 upregulated (FDR ≤ 0.05 and LFC ≥ 1.0) and 20 downregulated (FDR ≤ 0.05 and LFC ≤ -1.0) genes at day 3 *vs* 0, 276 upregulated and 153 downregulated genes at day 15 *vs* 3, and 16 upregulated and 2 downregulated genes at day 15 *vs* 0 (**Supplemental Figure 3**).

**Figure 6:**
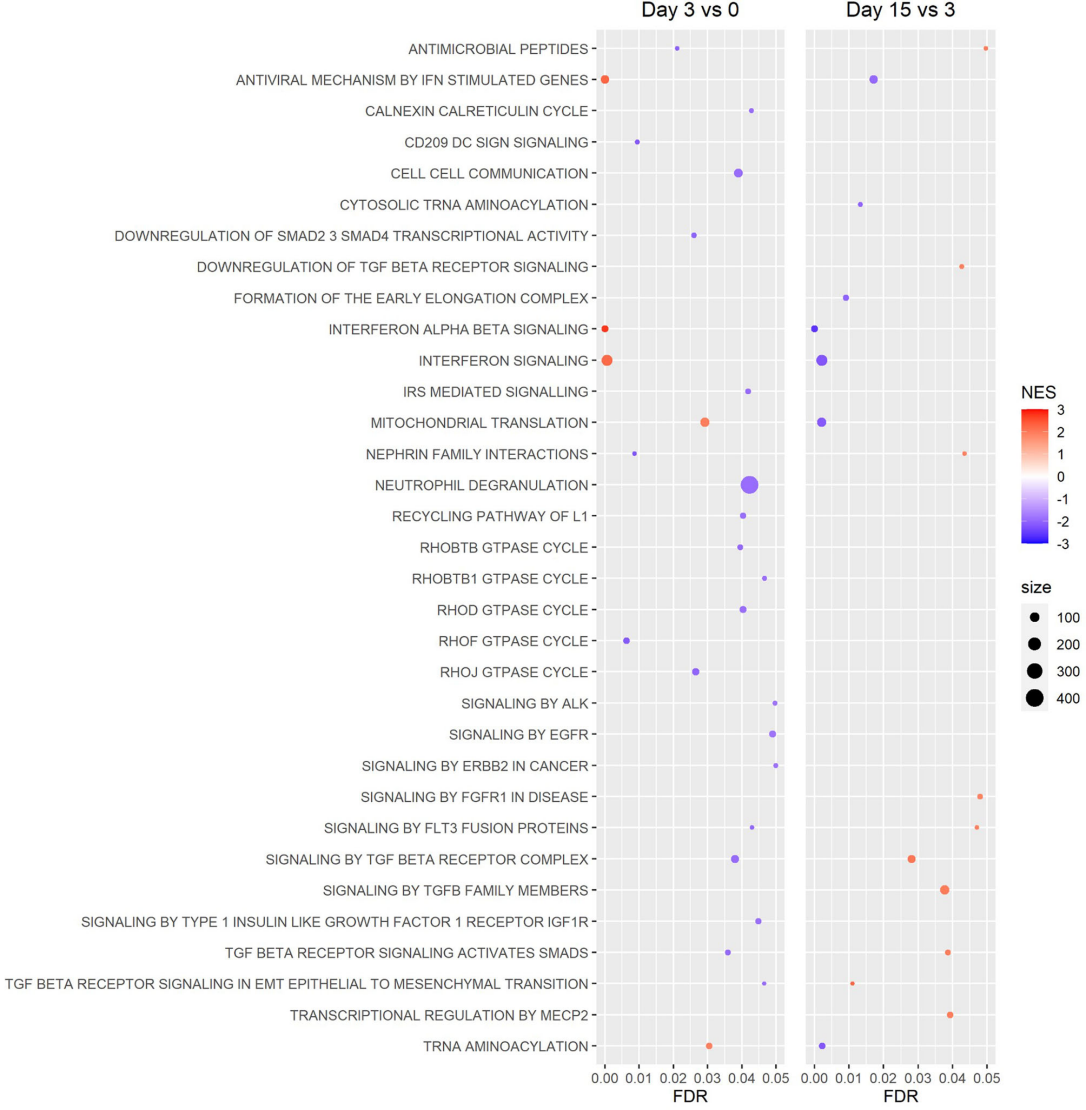
Gene Set Enrichment Analysis (GSEA) of Reactome canonical pathways. Gene sets revealed a pattern of increased expression of genes associated with interferon signaling and antiviral mechanisms between the beginning and peak of infection (day 3 vs 0), then decreased expression of these genes between the peak and resolution of infection (day 15 vs 3). No figure was included for the day 15 vs 0 comparison because no gene sets were significantly enriched. (NES: Normalized Enrichment Score).

**Figure 7:**
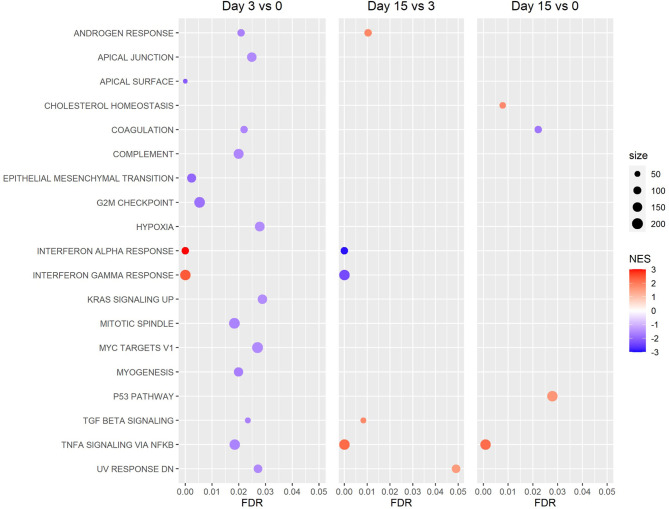
Gene Set Enrichment Analysis (GSEA) of Hallmark gene sets. Revealed a pattern of increased expression of genes associated with the interferon alpha and gamma response between the beginning and peak of infection (day 3 vs 0), then decreased expression of these genes between the peak and resolution of infection (day 15 vs 3). (NES: Normalized Enrichment Score).

GSEA of Reactome canonical pathway gene sets found several significantly enriched gene sets for the day 3 *vs* 0 and 15 *vs* 3 comparisons, while no significantly enriched gene sets were detected for the day 15 *vs* 0 comparison (**Figure 6**). Among the most significantly enriched gene sets were “interferon alpha beta signaling,” “interferon signaling,” and “antiviral mechanism by IFN stimulated genes,” all of which had a positive normalized enrichment score (NES) for the day 3 *vs* 0 comparison (NES ≥ 2.21), and a negative NES for the day 15 *vs* 3 comparison (NES ≤ -1.95) (**Figure 6**). GSEA of hallmark gene sets produced similar results, with the gene sets “interferon alpha response” and “interferon gamma response” being two of the most significantly enriched gene sets for the day 3 *vs* 0 and 15 *vs* 3 comparisons (**Figure 7**). Both the IFN alpha response and IFN gamma response hallmark gene sets had a positive normalized enrichment score (NES) for the day 3 *vs* 0 comparison (NES ≥ 2.4), and a negative NES for the day 15 *vs* 3 comparison (NES ≤ -2.3) (**Figure 7**). The Reactome “antiviral mechanism by IFN stimulated genes” gene set and hallmark “interferon alpha response” and “interferon gamma response” gene sets share several of the same highly significant DEGs, including IFIT1, IFIT3, ISG15, MX1, OAS1, and OASL, which are significantly upregulated (FDR ≤ 0.05 and LFC ≥ 1.0) at day 3 *vs* 0 and significantly downregulated (FDR ≤ 0.05 and LFC ≤ -1.0) at day 15 *vs* 3 (**Supplemental Figures 4–6**).

**Figure 8:**
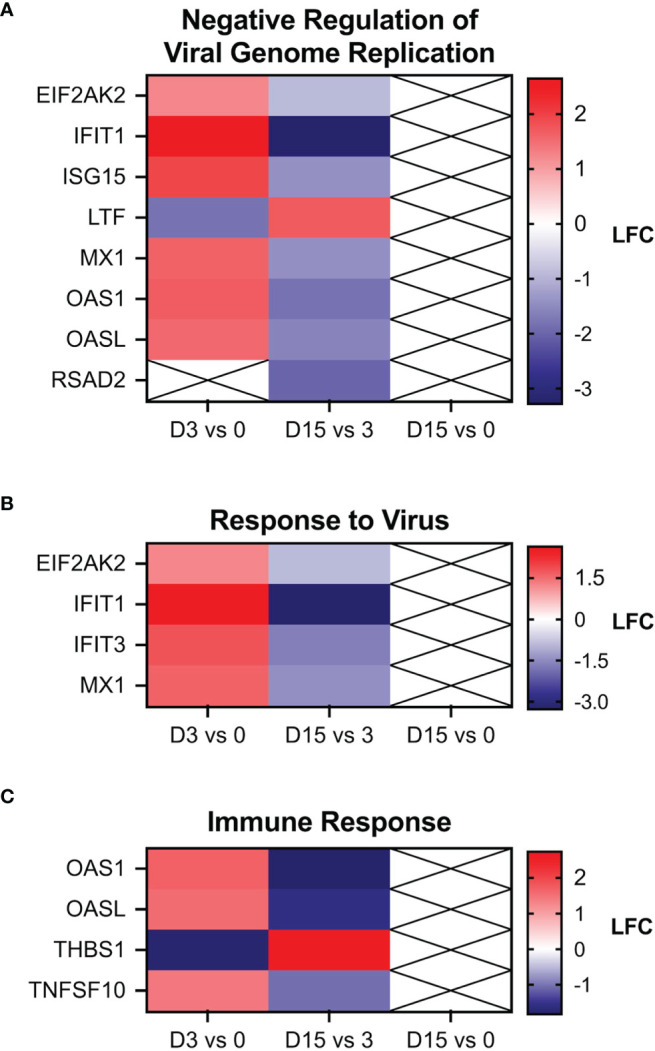
Heat maps of genes associated with the gene ontology (GO) terms. **(A)** “negative regulation of viral genome replication,” **(B)** “response to virus,” and **(C)** “immune response”. The three GO terms shown here were the only ones found to be significantly enriched by the DAVID Functional Annotation tool. No GO terms were found to be enriched for the day 15 vs 0 comparison. Boxes with an X indicates an FDR > 0.05.

DAVID GO analyses detected one significantly enriched GO term (GO:0045071, “negative regulation of viral genome replication”) associated with both the day 3 *vs* 0 and 15 *vs* 3 comparisons, and two additional GO terms that were significantly enriched for only the day 3 *vs* 0 comparison (GO:0009615, “response to virus” and GO:0006955, “immune response”). Heatmaps generated from the LFC values of DEGs associated with these terms indicate that these DEGs tend to be significantly upregulated (FDR ≤ 0.05 and LFC ≥ 1.0) at day 3 *vs* 0 and significantly downregulated (FDR ≤ 0.05 and LFC ≤ -1.0) at day 15 *vs* 3 (**Figure 8**). The transcriptomic data confirms the early detection of systemic interferon production presented in **Figure 3** and demonstrate to be a very sensitive technique for identifying changes associated with a transient, short-lived innate antiviral response to a subclinical ZIKV infection.

## Discussion

The baboon represents a NHP that has been frequently employed in biomedical research of viral diseases (Locher et al., 2003; Perry et al., 2012; Welliver et al., 2020; Singh et al., 2021). The baboon may also represent one of the sylvatic reservoirs of ZIKV, considering the baboon’s wide distribution throughout Africa and serological evidence of baboon ZIKV infection in the wild (Buechler et al., 2017). Consequently, the baboon has been recently explored as a NHP model for experimental ZIKV infection and pathogenesis (Gurung et al., 2018; Gurung et al., 2019; Peregrine et al., 2019; Gurung et al., 2020). In this study, we sought to increase validation of the baboon model of ZIKV infection by employing molecular techniques and high dimensional immunological analyses that have not been explored in published studies and that are frequently used in human clinical work or with other NHP species.

Whereas some baboon studies have seen clinical signs of ZIKV infection (Gurung et al., 2018; Gurung et al., 2019; Peregrine et al., 2019), we have seen an outcome similar to the more than 80% of human cases, with ZIKV infection resulting in a subclinical asymptomatic infection (Fauci and Morens, 2016; Jimenez et al., 2017; Haby et al., 2018). This different outcome between our studies and other baboon ZIKV studies may be explained by the use of a different infecting doses [10^4-6^ TCID_50_ in this study *vs* 10^6^ FFU in other studies (Gurung et al., 2018; Peregrine et al., 2019; Gurung et al., 2020)], differences in the infecting ZIKV strain [ZIKV Puerto Rico isolate in our case *vs* French Polynesia (Gurung et al., 2018; Peregrine et al., 2019)], or the sex and age of baboons [juvenile males in our study *vs* adult male or females (Gurung et al., 2018; Gurung et al., 2019; Peregrine et al., 2019)]. As for the effect of ZIKV infectious dose, we observed that the 10^6^ TCID_50_ infecting dose resulted in viremia in as short a time as 24 hr and early interferon response, but the duration and resolution of infection and immune response of the animals were not different from the outcome observed for animals receiving 10^4^ TCID_50_.

The flow cytometry and cytokine analyses of baboon blood after ZIKV infection showed the expected early activation of the innate immune response, which in turn resulted in activation of cells of the adaptive immune system. An interesting observation was the transient peak of expression of CD154 on CD4 T cells, considering that expression of CD154 is tightly regulated and occurs after T cell receptor engagement with an antigen-presenting cell; it is highly unlikely that all these CD4 T cells expressing CD154 were specific for ZIKV antigens, which could suggest some bystander activation of CD4 T cells, but additional studies would be needed to explain this phenomenon. The baboon pattern of early and transient systemic cytokine expression has also been seen in macaque models of ZIKV infection (Hirsch et al., 2017; Pantoja et al., 2017). Transcriptomic profiling of the baboon response to ZIKV infection revealed upregulation of genes associated with IFN alpha, beta, and gamma signaling and antiviral responses between days 0 and 3, then downregulation of the same sets of genes between days 3 and 15, a period when viral infection was effectively controlled. The upregulation of genes related to IFN signaling early in infection has been observed in other studies of ZIKV infection in whole blood of rhesus and cynomolgus macaques (Schouest et al., 2020), as well as in human PBMCs *in vitro* (Lim et al., 2020). Several genes found to be upregulated in both the Lim 2020 and Schouest 2020 studies were also upregulated (FDR ≤ 0.05 and LFC ≥ 1.0) in our results, including IFI44, IFIT1, IFIT3, MX1, OAS1, and OASL (**Supplemental Figures 4–6**). Finally, this study demonstrated that subclinical ZIKV infection can induce a protective immune response against re-exposure with an evolutionary distant ZIKV isolate, and that re-exposure to ZIKV induces an anamnestic immune response to the virus, even in the absence of detectable viremia, a finding that has also been reported in rhesus macaque models of ZIKV infection (Osuna et al., 2016; Schouest et al., 2020; Schouest et al., 2021).

The low number of replicates (*n* = 4 for immunological assays and *n* = 3 for RNA-seq) may lead to reduced statistical power and an increased margin of error, however, low numbers of replicates are relatively common among NHP studies due to economic and ethical considerations. Some ZIKV studies utilizing NHP models have been performed on cohorts of as few as 2 animals (Koide et al., 2016; Hirsch et al., 2017). Despite the limitation of sample size, NHP models are crucial to translational research, especially in the case of studying viruses such as ZIKV that do not naturally replicate in the cells of other commonly used model organisms, such as rodents (Nazerai et al., 2019).

In summary, the results of the molecular and omic techniques presented in this manuscript are similar to what has been learned with other macaque models of ZIKV infection and from human cases, and therefore validate these techniques when applied to the baboon ZIKV model. The baboon model of ZIKV infection offers a valid alternative to the macaque model and could alleviate the nationwide shortage of macaques available for biomedical research, which has been dramatically strained by the extensive use of macaques for COVID-19 studies (Chang et al., 2021). Besides availability, the baboon as an animal model stands on its own for studies on eclampsia (Carter, 2007), placentation (Carter, 2007), endometriosis (Kyama et al., 2007), neonatal development (Giavedoni et al., 2004; Warfel et al., 2014; Kuo et al., 2017), and maternal nutrition and offspring health (Li et al., 2017; Light et al., 2018). All these areas of study are still relevant for unresolved issues associated with ZIKV and complications with infection, and the molecular tools presented in this manuscript can contribute to their understanding.

## Data Availability Statement

The data presented in the study are deposited in the Sequence Read Archive (SRA) repository, accession number PRJNA811242.

## Ethics Statement

The animal study was reviewed and approved by Texas Biomedical Research Institute Institutional Animal Care and Use Committee.

## Author Contributions

LG contributed to conception and design of the study. VH, JC, LP, and V-OP performed experiments and acquired data. EC and PF directed the animal work. EM, JG, LC, and LG analyzed the data. EM and LG contributed to data visualization. LG, LC, JP, PF, and JG contributed resources and analysis tools. LG and EM wrote the manuscript. All authors contributed to the article and approved the submitted version.

## Funding

This investigation used resources that were supported by the Southwest National Primate Research Center grant P51 OD011133 from the Office of Research Infrastructure Programs, National Institutes of Health. Additional funding was provided by a SNPRC Pilot Project Program.

## Conflict of Interest

The authors declare that the research was conducted in the absence of any commercial or financial relationships that could be construed as a potential conflict of interest.

## Publisher’s Note

All claims expressed in this article are solely those of the authors and do not necessarily represent those of their affiliated organizations, or those of the publisher, the editors and the reviewers. Any product that may be evaluated in this article, or claim that may be made by its manufacturer, is not guaranteed or endorsed by the publisher.
