# Supplementary material for: Molecular Approaches for the Validation of the Baboon as a Nonhuman Primate Model for the Study of Zika Virus Infection
Source: Front Cell Infect Microbiol. 2022 Apr 14;12:880860. doi: 10.3389/fcimb.2022.880860 (PMC9046911; doi:10.3389/fcimb.2022.880860)
Supplement: Supplementary file 1 [file Table_1.docx]

Supplementary Material

| **A.** | 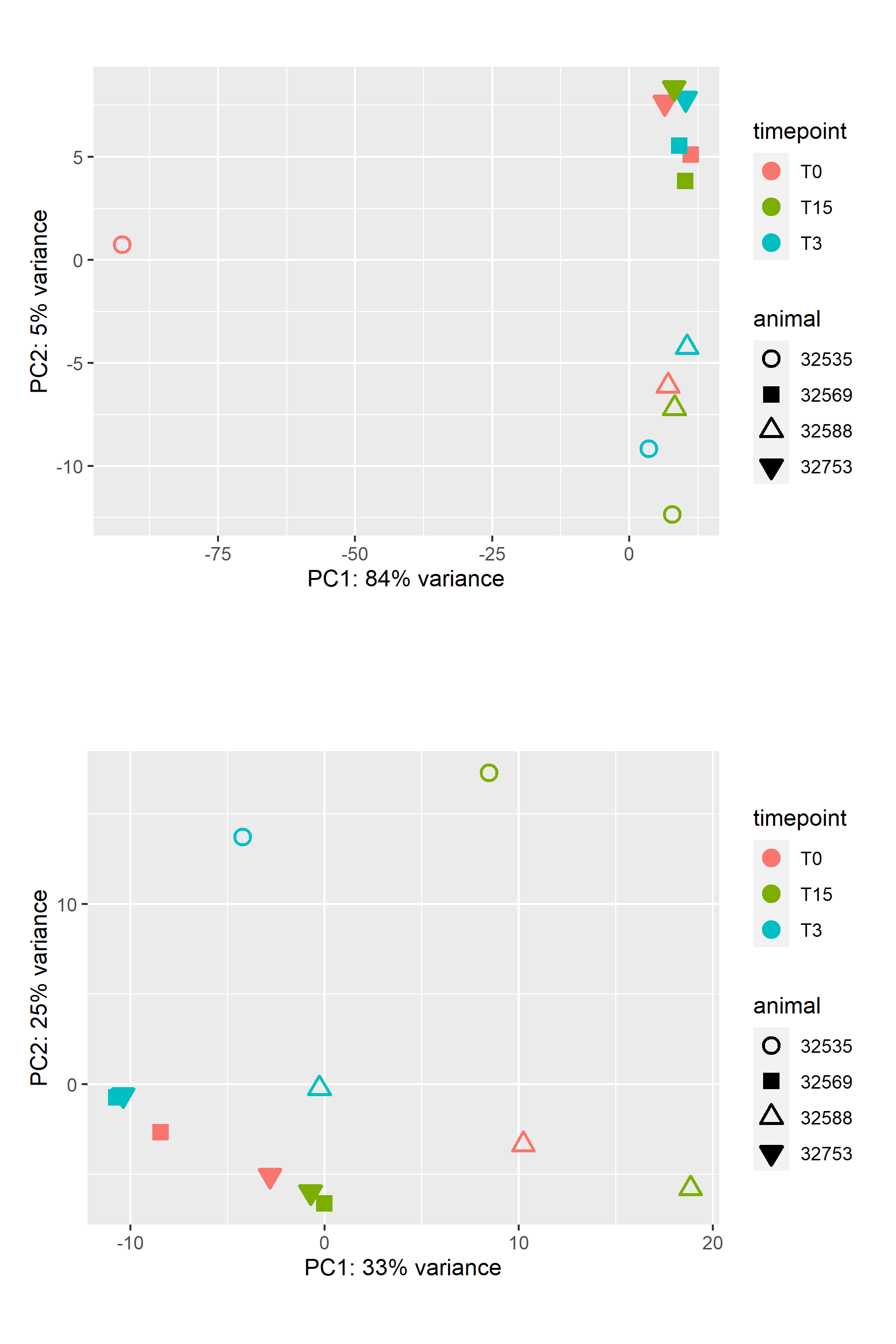 |
| --- | --- |
| **B.** | 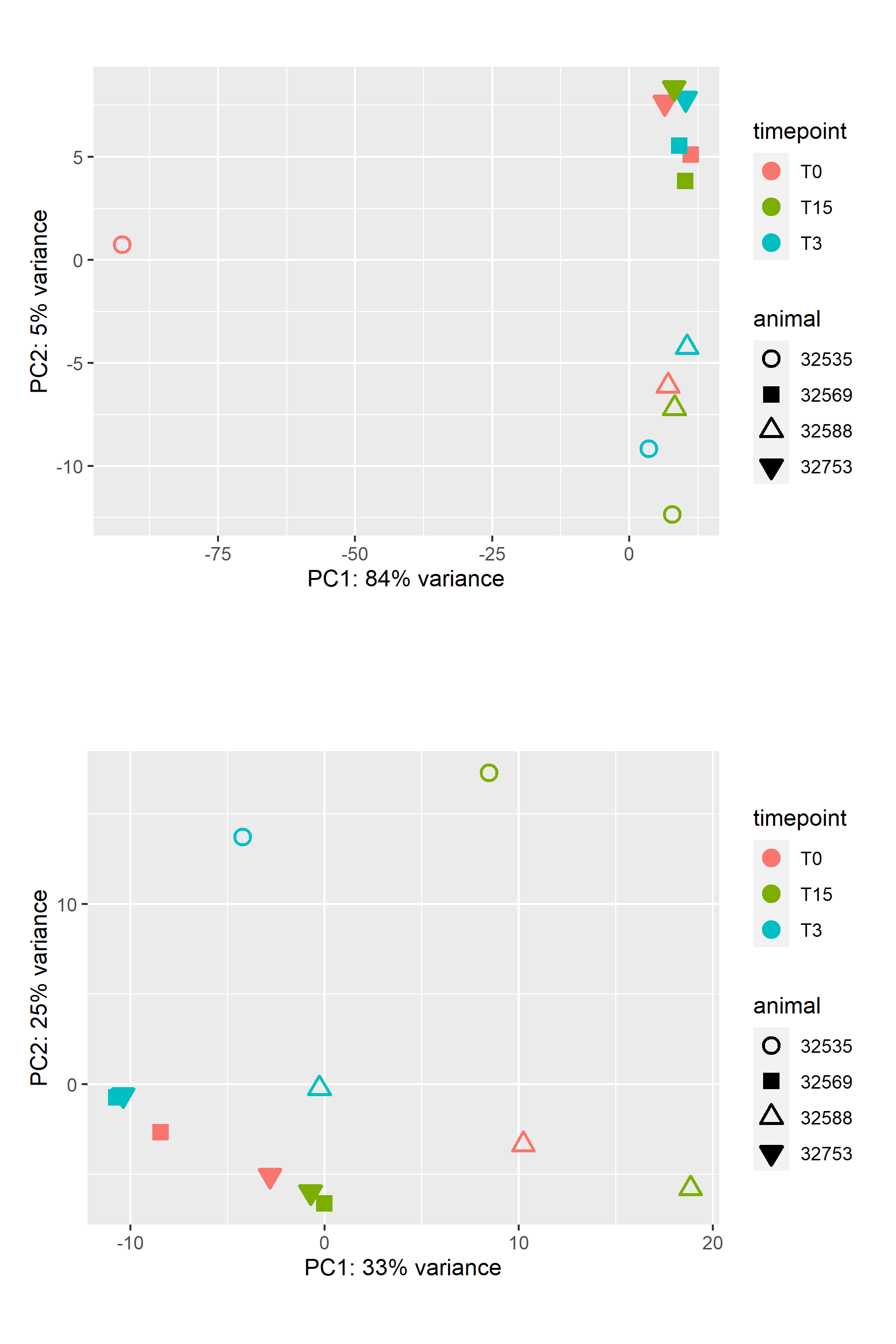 |

**Supplemental Figure 1.** Principal component analysis (PCA) plots of gene counts normalized by variance stabilizing transformation (VST). **(A)** PCA plot including all twelve samples (from four animals at three timepoints) indicating that animal 32535 is an outlier at the day of infection (D0). **(B)** PCA plot with all samples except animal 32535 at D0, suggesting that animal 32535 was also an outlier at the other two timepoints (D3 and D15). All data from animal 32535 was excluded from downstream analyses.


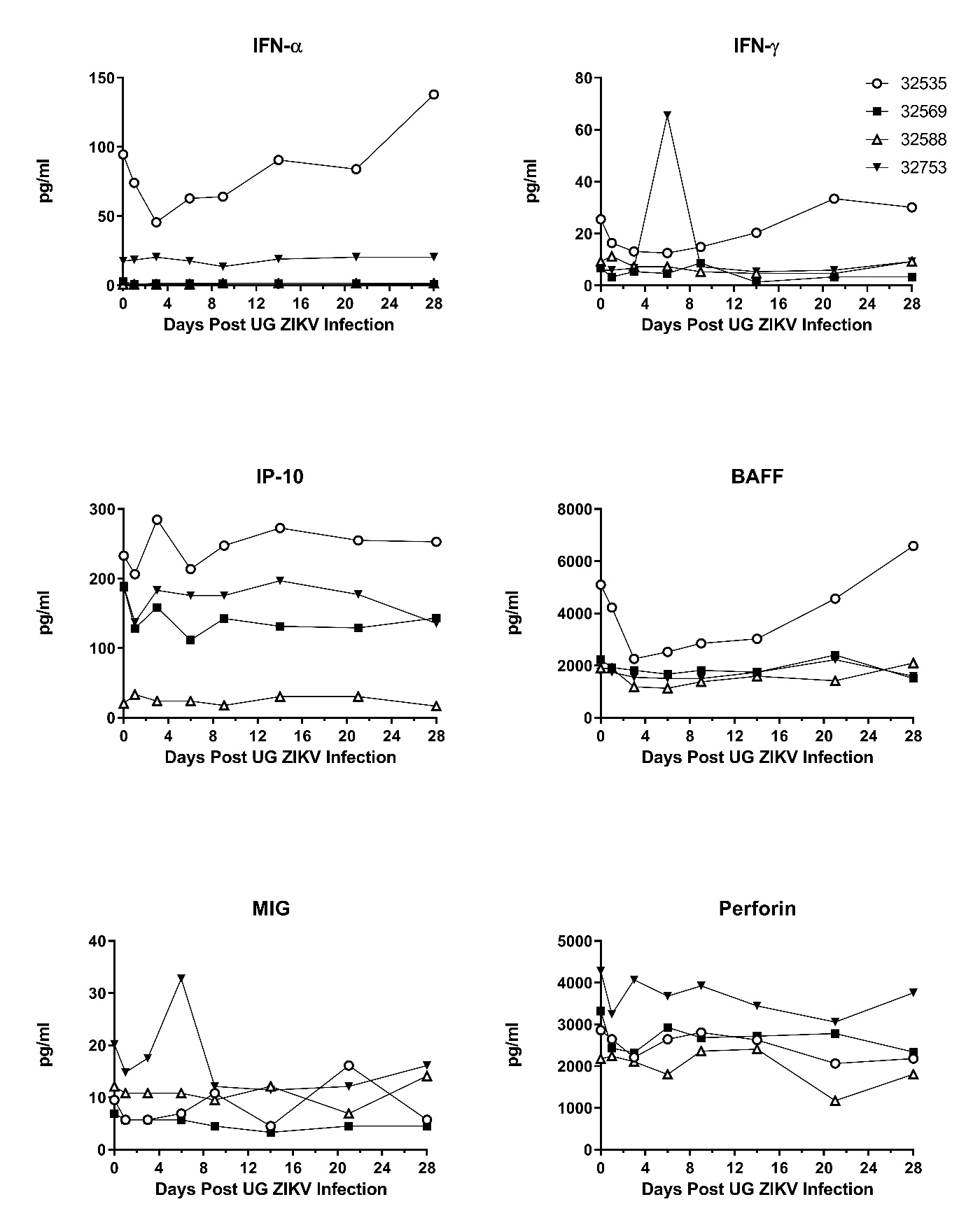


**Supplemental Figure 2.** Baboon plasma cytokine levels after ZIKV UG infection. (A) IFN-α, (B) IFN-γ, (C) IP-10/CXCL10, (D) BAFF, (E) MIG/CXCL9, and (F) perforin. Cytokines were measured using Luminex assays. The baboons were challenged with the virus in either a high dose of 10^6^ TCID_50_ (*n* = 2, open symbols) or a low dose of 10^4^ TCID_50_ (*n* = 2, closed symbols).


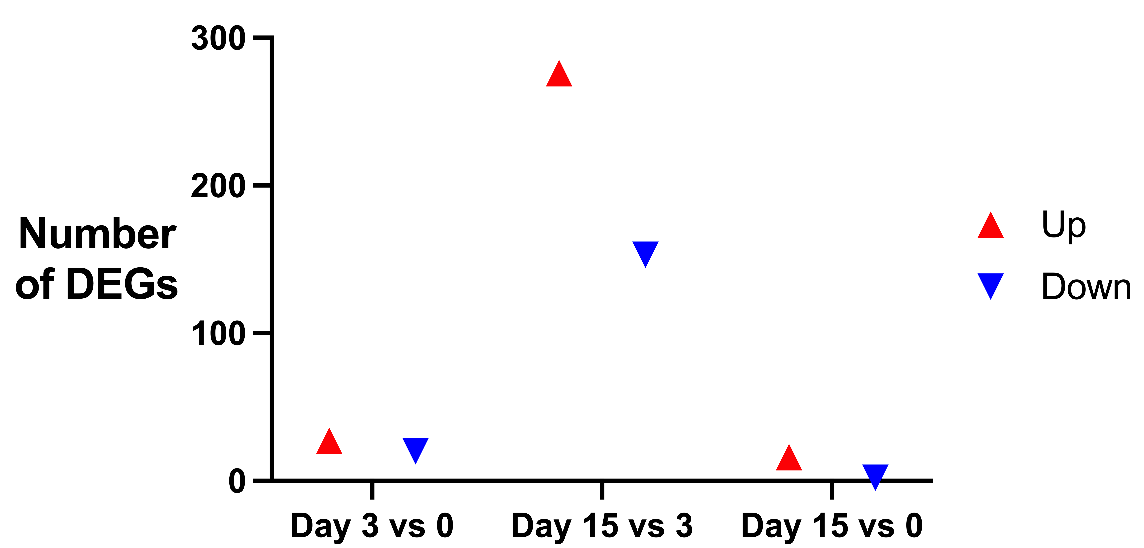


**Supplemental Figure 3.** Number of differentially-expressed genes (FDR ≤ 0.05 and LFC ≥ ±1.0) detected by Wald test pairwise comparisons of days 3 vs 0, 15 vs 3, and 15 vs 0 post infection (*n* = 3).

| **A.** | 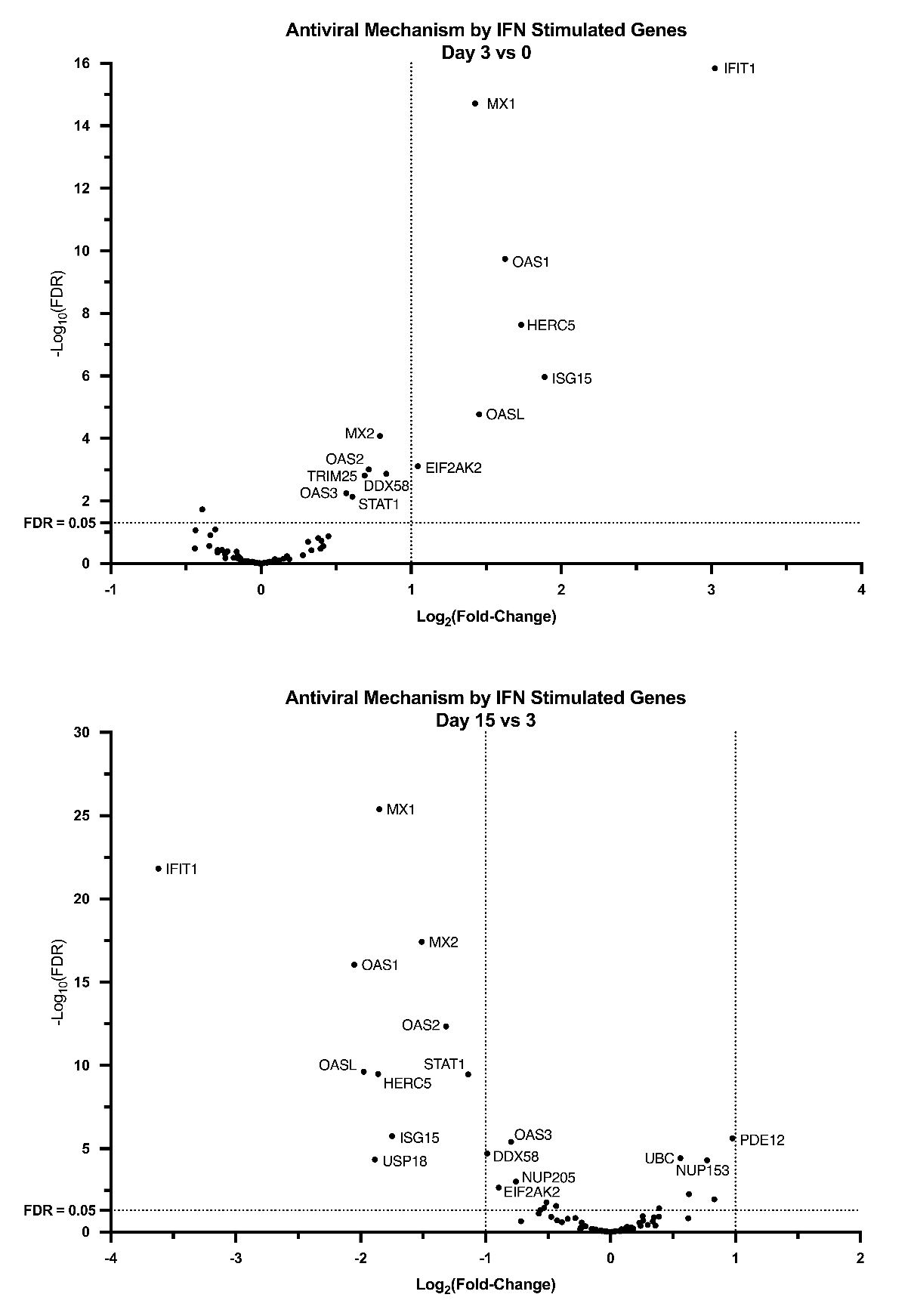 |
| --- | --- |
| **B.** | 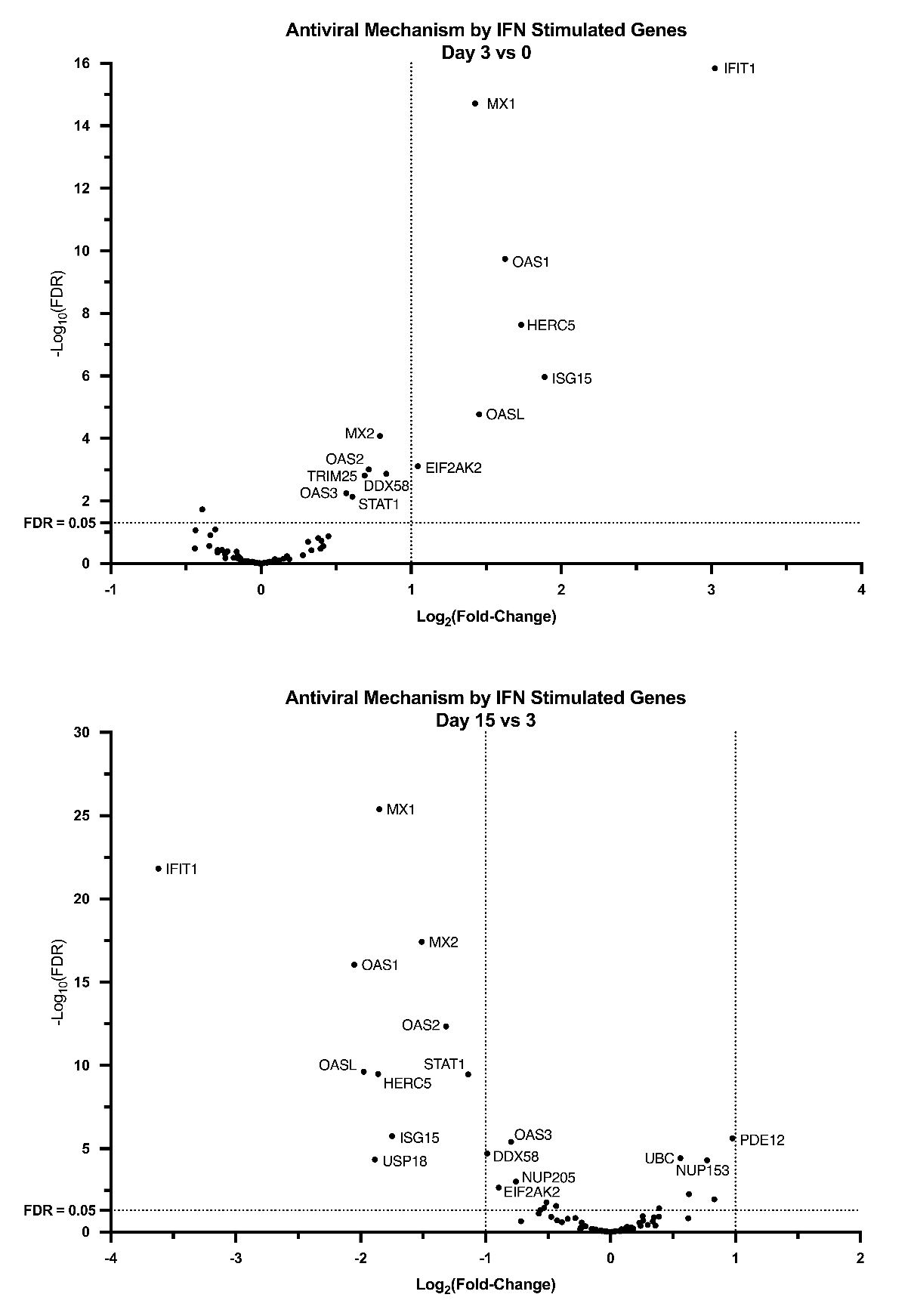 |

**Supplemental Figure 4**. Volcano plots of genes associated with the Reactome gene set “antiviral mechanism by interferon stimulated genes”. **(A)** increased expression between days 0 and 3, then **(B)** decreased expression between days 3 and 15. No figure was included for the day 15 vs 0 comparison because the gene set was not significantly enriched for that comparison.

| **A.** | 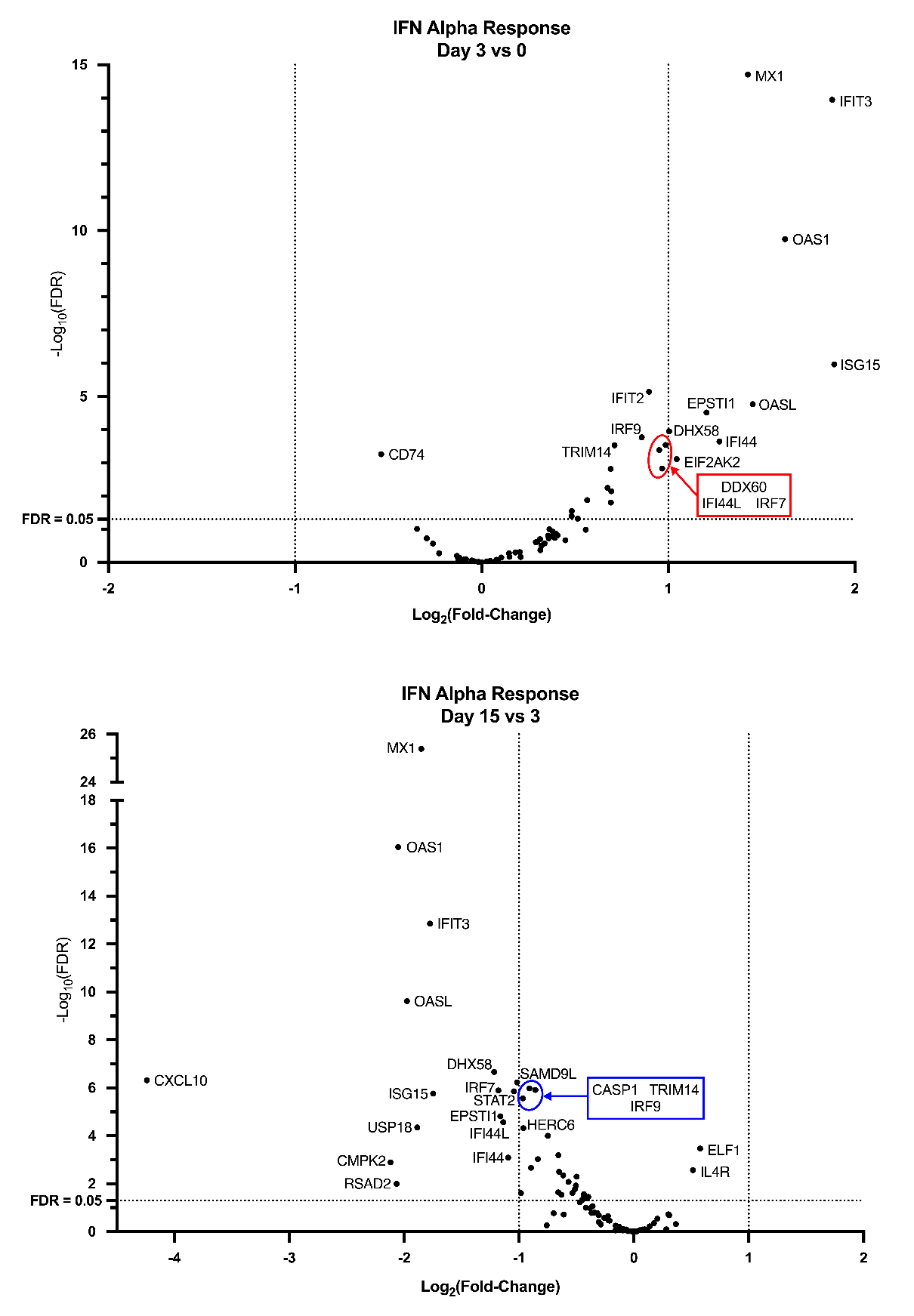 |
| --- | --- |
| **B.** | 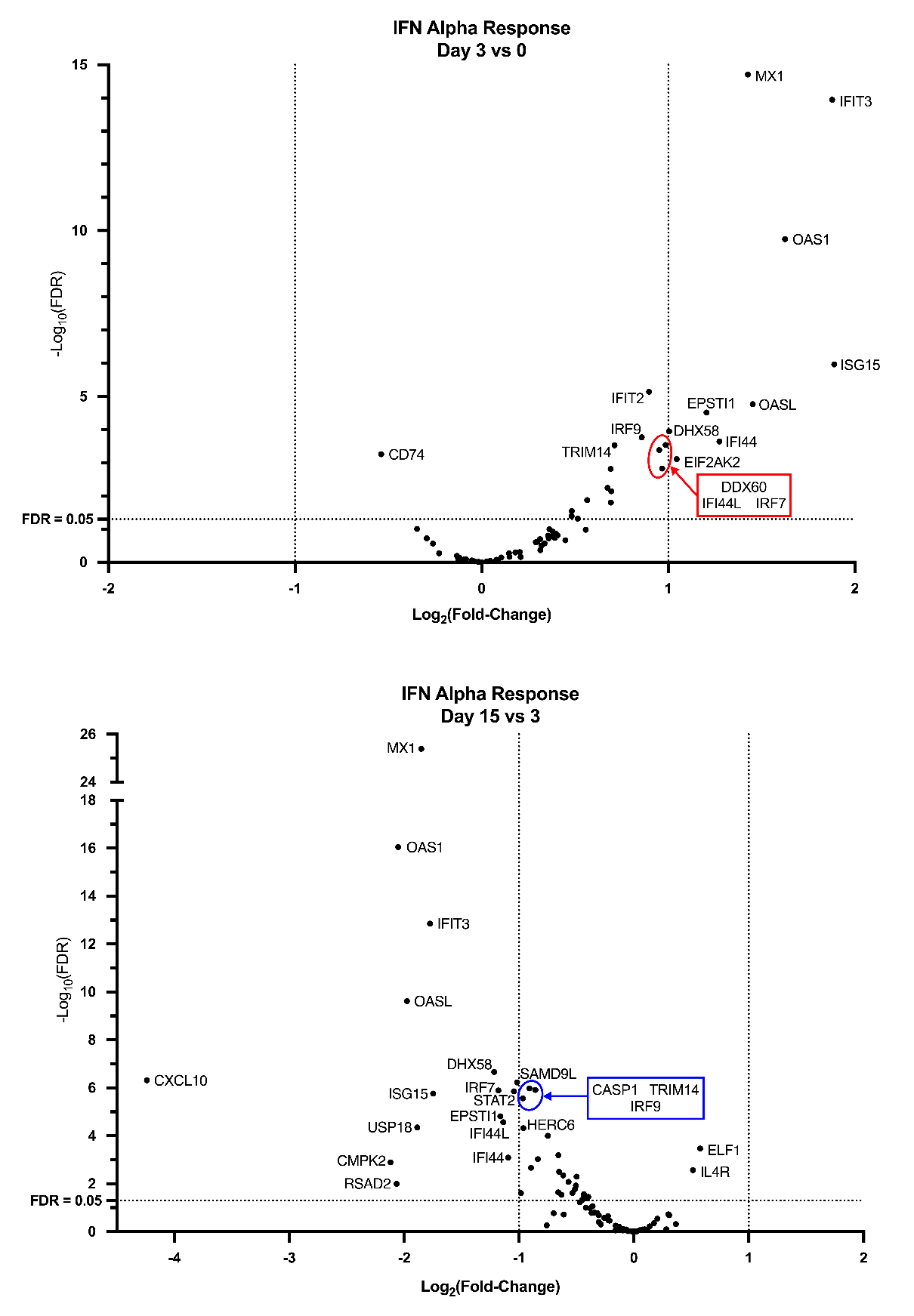 |

**Supplemental Figure 5**. Volcano plots of genes associated with the Hallmark gene set “interferon alpha response”. **(A)** increased expression between days 0 and 3. **(B)** decreased expression between days 3 and 15. No figure was included for the day 15 vs 0 comparison because the gene set was not significantly enriched for that comparison.

| **A.** | 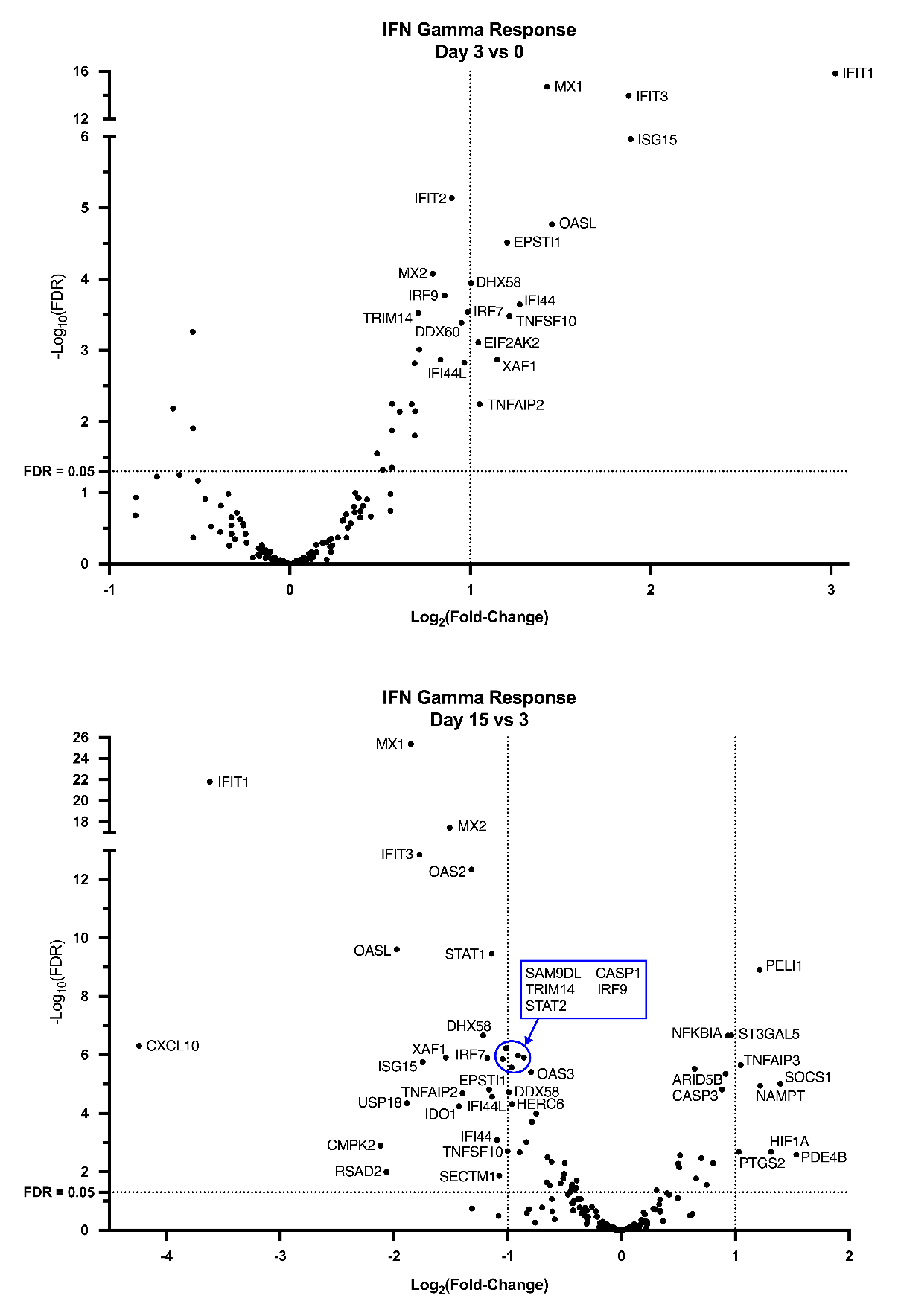 |
| --- | --- |
| **B.** | 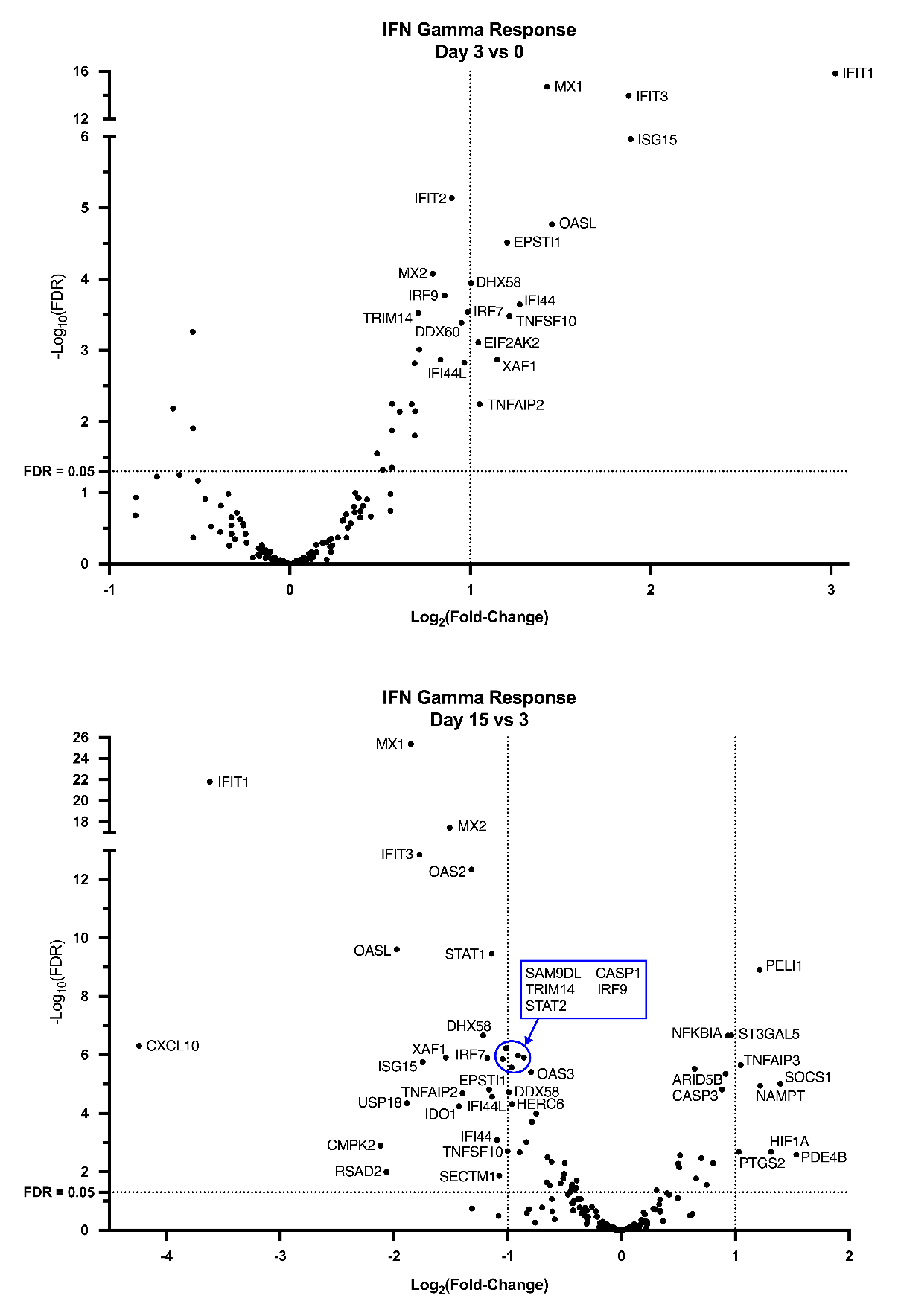 |

**Supplemental Figure 6**. Volcano plots of genes associated with the Hallmark gene set “interferon gamma response”. **(A)** increased expression between days 0 and 3. **(B)** decreased expression between days 3 and 15. No figure was included for the day 15 vs 0 comparison because the gene set was not significantly enriched for that comparison.
